# The effects of a psychiatric clerkship on stigmatizing attitudes toward mental disorders as held by German medical students

**DOI:** 10.3389/fpsyt.2023.1306403

**Published:** 2023-12-08

**Authors:** Maxim Zavorotnyy, Simon Klatte, Yunbo Yang, Wei Liu, Ulrich Wagner, Tilo Kircher

**Affiliations:** ^1^Department of Psychiatry and Psychotherapy, Psychiatric Services Aargau, Academic Hospital of the University of Zurich, Windisch, Switzerland; ^2^Department of Psychiatry and Psychotherapy, University of Marburg, Marburg, Germany; ^3^Department of Experimental Psychopathology, Institute of Psychology, University of Hildesheim, Hildesheim, Germany; ^4^Department of Social Psychology, University of Marburg, Marburg, Germany

**Keywords:** Germany, mental disorders, psychiatry, social stigma, stereotyping, undergraduate medical education, vulnerable populations, clinical clerkship

## Abstract

**Background:**

According to the United Nations, access to medical care is a fundamental human right. However, there is widespread stigmatization of severe mental illnesses and this appears to seriously hamper the quality of healthcare in people with psychiatric co-morbidity. Thus, interventions that help reduce stigma among healthcare providers are urgently needed.

**Purpose:**

The objective of the current study was to investigate the effects of a psychiatric clerkship on stigmatizing attitudes toward mental disorders held by medical students.

**Methods:**

Between 2018 and 2019, a total of 256 third- and fourth-year students from Marburg University Medical School (Germany) completed two surveys—one before and one after a 2 week clerkship program that was designed to prioritize direct interaction with the patients. For measuring stigma, the questionnaires contained questions about students' attitudes toward psychiatry (ATP), including the Opening Minds Scale for Healthcare Providers (OMS-HC), Community Attitudes Toward the Mentally Ill (CAMI), and measurements according to the Stereotype-Content Model (SCM). We conducted pre-vs.-post comparisons using the Wilcoxon signed rank test with continuity correction or paired *t*-test and employed the Spearman method for correlational analysis. We considered *p* < 0.05 significant and adjusted all *p*-values reported here using the Benjamini-Hochberg procedure to account for family-wise error.

**Results:**

After the clerkship, a significantly reduced stigma was found, as assessed with ATP (mean *p* < 0.001), OMS-HC (sum and subscale “attitudes” *p* < 0.001; subscale “disclosure” *p* = 0.002), and both SCM subscales (*p* < 0.001). Moreover, we observed significant associations between stigma expression (e.g., OMS-HC sum) and the willingness of students to choose psychiatric residency after finishing medical school (before clerkship: *p* < 0.001; *ρ* = −0.35; change after clerkship: *p* = 0.004; *ρ* = −0.2).

**Conclusion:**

Our findings indicate that a psychiatric clerkship that involves students in direct interaction with patients may effectively reduce stigma. Therefore, we advocate the incorporation of components of direct interaction in medical education to combat stigma and unequal treatment, as this could improve outcomes in patients with severe mental illnesses.

## 1 Introduction

Access to medical care is recognized by the United Nations as a fundamental human right (1) and should therefore be provided to all groups of patients in accordance with general standards, and must avoid systematic discrimination against minoritized populations. However, there is a dramatic mortality gap in people with severe mental illness in high-income countries (2, 3) and this cannot only be explained by factors related to the mental illness itself or its treatment, or to the patients' lifestyle. Besides all these factors, this mortality gap must, at least in part, be a consequence of the difficulties that people with mental illness experience in accessing appropriate health care and may be associated with stigmatization by healthcare professionals (4, 5). Furthermore, social stigmatization can be a significant barrier to suicide prevention, particularly in low- to middle-income countries (6).

According to international studies, psychiatric disorders are estimated to have a lifetime prevalence of ~30% and a 12-month prevalence of 17.6% (7). At the national level in 2010, mental conditions were Germany's fourth most prevalent disease group in terms of disability-adjusted life years (8). At the same time, misinformation and misconceptions about stigmatizing attitudes to psychiatric illnesses prevail in the population [e.g., (9)]. Since stigmatization of people with mental illness is both a risk factor and a consequence of mental illness, it seems to be a significant obstacle to seeking help for recovery. Thus, mechanisms that help reduce this stigmatization need to be systematically studied. Effective mechanisms should be implemented in our daily practice as a matter of urgency.

### 1.1 Stigma among healthcare provides

The phenomena of stigmatization do not only affect the general population and people with mental illness, but are also prevalent among healthcare providers such as physicians or medical students, social workers, and nursing staff, whether they work in somatic or psychiatric care (10). For example, there is a less willingness to treat people with mental illness, especially those diagnosed with schizophrenia, due to concerns about an increased predisposition to violence (11–14). Additionally, working in psychiatry is often considered by medical students unsatisfying and stressful (15). Therefore, expressions such as “emotionally stressful,” “overwhelming,” “clerkship with mentally disturbing images,” or “working with crazy people makes you crazy” are widely used in evaluations of psychiatric training among medical students (15). Although psychiatry does not have a bad reputation among medical students, they are less likely to opt for post-residence training in psychiatry, and this leads to a shortage of young psychiatric staff (11, 15–17). Possible reasons include fear of criticism from family and friends, due to the negative image of psychiatry compared to other specialties, and a potential risk of being stigmatized as “neurotic” or “weird.” In addition, there is a misconception that psychiatry is unscientific and inaccurate (16).

Stigmatizing attitudes also seem to influence treatment-relevant decisions. For example, the recommendation of a weight reduction program was not even made in patients with schizophrenia, mainly due to the preconception of reduced motivation, difficulties in information intake, and reduced personal responsibility (18). Additionally, the false attribution of physical symptoms to a mental condition—also known as “diagnostic overshadowing”—is also a significant medical problem attributable to stigma (19). Not unexpectedly, a large body of evidence shows that stigma among medical providers seriously decreases the quality of care (5). However, there are promising retrospective data that demonstrate that stigma in medical providers has tended to decrease over the past 30 years (20). Furthermore, previous research indicates that stigma expression can depend on individual characteristics and professional education. For example, in an evaluation using a case vignette (21), the level of stigma and the tendency to greater social distance were lower in students who reported previous personal contact with people with mental illness and in those who had participated in relevant professional training in healthcare.

### 1.2 Role of psychiatry training in stigma reduction

A clerkship in psychiatry is an essential part of medical training. In addition to teaching specialized knowledge and communication skills, the reduction in stigma toward mental illness is a necessary goal of the psychiatric degree program (11, 13, 17). Personal contact with people with mental illness seems crucial in helping to reduce stigma (22–24). The importance of personal contact has already been demonstrated, for example, by its favorable effects on attitudes toward the benefits of psychopharmacology, prognosis, and explainability of mental illness (25). In addition to personal contact, the length and quality of the clerkship appear to influence stigma reduction (26). Furthermore, the motivation of the practitioner or teacher—as a role model—must be mentioned as a further relevant influencing factor (27).

In general, most studies show a reduction in stigma after a psychiatric clerkship (16). However, there are reports of unaffected and even increased stigma after a psychiatric clerkship (11, 28–30). Aside from methodological and intercultural aspects, these conflicting results may be associated with differences in the way students perceived their trainers and hospital staff, and in how the clerkship was organized, (31, 32). Furthermore, it could be assumed that the participation of students in direct patient interaction during the curricular program may differ in different medical school programs. However, there is emerging evidence that shows that contact between members of different groups leads to a significant reduction in prejudice (33, 34), as first postulated by Gordon Allport and known as the intergroup contact theory (35).

### 1.3 Rationale for the study and hypotheses

Since a direct interaction between students and patients is an essential compound of the psychiatric clerkship at the Marburg University Medical School, the objective of the current study was to evaluate our program in terms of its effectiveness in reducing stigma in the context of intergroup interaction. For this purpose, we included the measurement according to the stereotype-content model (36), that recently demonstrated its reliability in testing stigmatizing stereotypes between groups [e.g., (37)]. As our *a priori* hypothesis, we assumed a reduction in stigma after our clerkship in psychiatry.

Given the increasing need and current shortage of mental health professionals, we were interested to see how stigma and its change after the clerkship influence the desire to become a psychiatrist in the future. Therefore, we hypothesize that a high level of expressed stigma is associated with a lower willingness to complete specialist training in psychiatry after finishing medical school. Additionally, we assumed that a stronger increase in willingness to complete specialist psychiatry training is associated with a more prominent reduction in stigma.

## 2 Materials and methods

We conducted an interventional cohort study using opinion surveys. Initially (T1) and after completing (T2) the psychiatric clerkship, medical students were asked to answer a multiple choice questionnaire. The item count varied between baseline ($N_{\text{items}}^{\text{T1}}=80$) and follow-up due ($N_{\text{items}}^{\text{T2}}=76$) to the static nature of certain measures—e.g., Big-Five personality traits—, and the time-specific relevance of others such as post-clerkship student perceptions.

### 2.1 Participants

A total of 256 students completed the psychiatry clerkship in the summer semester of 2018 (*n* = 118) and the winter semester of 2018/19 (*n* = 138) and participated in the present study. For 73 subjects (29%), the survey data were incomplete in T1 or T2, so we excluded these cases as non-completers. Thus, 183 data sets were available for a completer analysis. All students were in their third and fourth year at the time of the study. **Table 2** provides an overview of our cohort.

### 2.2 Intervention

The clerkship in psychiatry—obligatory clinical part of the medical school curriculum—takes place between the sixth and tenth semesters (3rd and 5th year of medical school training), parallel with the other so called “head subjects” (e.g., neurology, otolaryngology, 1 week in psychosomatic medicine, and 1 week in child psychiatry). Together with psychiatry, the complete psychiatric-psychosomatic training block lasts four weeks. In the first or second pre-clinical year of study, students have two 1.5-h teaching sessions with patient videos and theoretical presentations of the psychopathological findings.

Our psychiatric internship spans the weekdays in two weeks. On average, students spent 8.8 of the scheduled ten days at the department. The internship is divided into a practical part from 8:00 to 11:30 a.m. on the ward and a theoretical part from 2:30 to 4:15 p.m. There are no classes during weekends or on holidays. One day of absence is allowed. Attendance is certified and is a prerequisite for taking the final exam and obtaining the certificate. At 8:00 a.m., the students attend the morning briefing, where the reports of previous inpatient admissions and the planning of the upcoming day occur. Twice a week, this meeting includes specific continuing education, case presentations, or journal clubs. Lectures with psychiatric topics can be attended from 11:30 a.m. to 2:30 p.m. In the afternoons, from 2:30 to 4:30 p.m., students participate in disease-specific seminars, including patient presentations and case discussions. Internal and external lecturers are responsible for the seminars. The external lecturers must be qualified as experienced therapists trained in medical didactics.

The students are divided into small groups of three to five participants and assigned to one of the six wards with different disease focuses, e.g., elderly psychiatry, depression, psychosis, anxiety and obsessive-compulsive disorders, addiction, or urgent psychiatry. Students spend the entire internship on the assigned wards and participate in their activities, including psychotherapy groups and individual sessions, as well as occupational or movement therapies. Each small group has an assigned academic mentor—a physician or psychologist. Furthermore, each student is assigned a patient with whom he or she conducts a medical history interview, accompanies during therapies, and writes a case discussion. During the training, students discuss different aspects of patient contact with their mentors. Finally, each student presents his or her case to the senior physician of the ward, who grades the student's work with the patient.

Successful completion of the clerkship requires fulfilling three criteria:

- sufficient attendance (defined as having <1 day of absence)
- accomplished presentation and discussion of the patient's case with the senior physician of the ward
- achieving a minimum score of 60% in the final multiple-choice test

The failure rate for the final multiple-choice examination varies from semester to semester but is typically below 5%. Overall, the psychiatry training is rated as average by students in the faculty ranking.

### 2.3 Procedure and recruitment

At the beginning of the study, the participating students received a subject information sheet and signed an informed consent form. Participants completed two anonymous questionnaires—before (T1) and after finishing the clerkship (T2). To identify the corresponding pairs of baseline and follow-up sheets, we instructed participants to generate a unique code that was used consistently on both questionnaires (T1 and T2). To avoid responses affected by conflicts of interest or social desirability, completion of the questionnaire was mandatory. The Marburg University Ethics Committee approved our study protocol.

### 2.4 Measurement tools

In the baseline questionnaire, we assessed the basic characteristics of participants (age, gender, semester assignment), whether or not the student had accomplished the clerkship in child psychiatry, and the previously experienced contact with people with mental disorders, employing the Level-of-Contact Report [LOCR; (38)]. We also briefly assessed the personality profiles using the Big Five personality traits model (39, 40). To evaluate the expression of stigma at baseline and after the clerkship, we asked the participants about their attitudes toward psychiatry (ATP) and used established measuring tools such as the Stereotype-Content Model [SCM; (36)], Opening Minds Scale for Health Care Providers [OMS-HC; (41)], and Community Attitudes Toward the Mentally Ill [CAMI; (42)]. Finally, we assessed the number of days the participants attended the clerkship and asked them to evaluate it. Table 1 provides an overview of the measured parameters and the time points. For a brief description of the measurement tools, see the Supplementary material.

**Table 1:** Measurement tools and time points.

**Measurement tools**	**Time points**	
	**T1**	**T2**
**Baseline only**		
Age, gender, semester assignment	+	−
Completed clerkship in child psychiatry	+	−
Big five personality traits	+	−
Level-of-contact report	+	−
**Baseline and follow-up**		
Attitude toward Psychiatry	+	+
Stereotype-content model	+	+
Opening minds scale for health care providers	+	+
Community attitudes toward the mentally ill	+	+
**Follow-up only**		
Number of days of clerkship	−	+
Clerkship evaluation questionnaire	−	+

### 2.5 Statistical analysis

For statistical analyzes, we used IBM^®^ SPSS^®^ Statistics 29 (43) and R software (44).

For the assumption of a normal distribution, we used the Shapiro-Wilk test and additionally considered density and quantile-quantile plots. To estimate the internal consistency of the scales used in our questionnaire, we applied the Cronbach α test.

To check for possible bias, we compared the group of “non-completers” (participants who completed the T1 questionnaire only) and “completers” (available T1 and T2 data), using the unpaired Wilcoxon rank sum test with continuity correction for categorical and metric-scaled variables if normal distribution cannot be assumed. For normally distributed variables, we performed the Student or Welch *t*-test. For comparisons of categorical variables, we used the χ^2^ test and, if required, the Fisher exact test.

To test our hypothesis of a reduction in stigma after the clerkship, we applied Student *t* or Wilcoxon rank sum paired tests to compare the SCM, OMS-HC, and CAMI values at visit T1 vs. T2. Since we assumed an effect of reduction, we performed these tests one-sided. This analysis was only conducted for the completers.

To test for the association between willingness to complete psychiatric specialist training and stigma, we performed a correlational analysis. Since the variable “willingness…” was categorical and we assumed directed effects, we applied one-sided Spearman rank correlations. Since the these data was available at baseline in completers and non-completers, this analysis was conducted for all participants.

To test our hypothesis of an association between increase in “willingness…” and reduction in stigma after clerkship, we calculated increments (Δ) representing changes in variables. Since high values of the items we tested here may represent higher or lower expression, depending on the scale, we calculated Δ using two distinct methods:

- For all variables testing the “attitude toward psychiatry”, the “stereotype-content model” as well as the CAMI subscales “benevolence” and “community mental health ideology,” we assumed that the values at T2 are higher than at T1. Therefore, we calculated increments using Formula 1.

(1) $$\begin{array}{l} \Delta ={\text{Value}}_{\text{T2}}-{\text{Value}}_{\text{T1}} \end{array}$$

- For all variables testing the “OMS-HC” as well as the CAMI subscales “authoritarianism” and “social restrictiveness”, we assumed that values at T1 are higher than at T2. Therefore, we calculated increments using Formula 2:

(2) $$\begin{array}{l} \Delta ={\text{Value}}_{\text{T1}}-{\text{Value}}_{\text{T2}} \end{array}$$

In the next step, we performed a further correlational analysis with the calculated increments (Δ) using the one-sided Spearman rank correlation for the completers only.

We assumed statistical significance at *p* < 0.05. However, since multiple tests were performed, we used the False Discovery Rate (FDR), according to the Benjamini-Hochberg procedure, to take the family-wise error (FWE) into account.

## 3 Results

All internal consistencies are adequate (α_Cronbach_ > 0.7) except for the subscale competence of the SCM (complete cohort at T1: SCM competence α = 0.68), OMS-HC attitudes (α > 0.6 and < 0.7); ATP (completers at T2: α = 0.59), OMS-HC disclosure (α < 0.6), CAMI authoritarianism (completers at the beginning of the study: α = 0.65), CAMI social restrictiveness (α < 0.6), and the CAMI community mental health ideology (α < 0.6).

### 3.1 Complete sample at baseline

A total of 256 medical students participated in the survey, 166 (65%) of whom were women. Forty-two percent of the participants (*n* = 106) had previously completed a child psychiatry clerkship. Sixteen participants (6%) reported having had a mental illness themselves (LOCR item 5), and 23 (9%) reported living with at least one person who had a mental illness (LOCR item 10). See Table 2 for more descriptive data. Although participants showed an average (Value = 3) personal interest (Mean = 3.20, [SD] = 0.92), their knowledge (2.08 [0.66]) and willingness to complete psychiatric specialist training (2.06 [1.13]) was below the average level at the start of the study. For the Stereotype-Content Model, the baseline means that the domains “warmth” (4.11 [0.83]) and “competence” (3.68 [0.82]) were also above average, corresponding to a moderate level of stigma, as measured with OMS-HC and CAMI (see Table 2).

**Table 2:** Descriptive statistics and comparisons between completers and non-completers at baseline (T1).

**Characteristic**	***N***	**Overall, *N* = 256^a^**	**Completers, *N* = 183^a^**	**Non-completers, *N* = 73^a^**	***p*-value^b^**	***q*-value^c^**
Sex	254				0.14	0.25
Male		166 (65)	124 (68)	42 (58)		
Female		88 (35)	58 (32)	30 (42)		
Age in years	254	24.36 (2.64)	24.40 (2.68)	24.25 (2.56)	0.77	0.77
Internship in child psychiatry	252	106 (42)	81 (45)	25 (35)	0.17	0.25
**Level-of-contact report**						
Sum	255	26 (13)	26 (13)	27 (14)	0.74	>0.99
Yes by LOCR_5	254	16 (6.3)	12 (6.6)	4 (5.5)	>0.99	>0.99
Yes by LOCR_10	254	23 (9.1)	16 (8.8)	7 (9.7)	0.82	>0.99
**Big five personality traits**						
Extraversion	255	−0.90 (2.67)	−1.03 (2.64)	−0.56 (2.72)	0.25	0.35
Agreeableness	255	1.99 (2.20)	2.10 (2.15)	1.71 (2.30)	0.16	0.35
Conscientiousness	255	−2.32 (2.25)	−2.51 (2.11)	−1.83 (2.51)	0.040	0.20
Neuroticism	255	2.24 (2.17)	2.30 (2.21)	2.10 (2.06)	0.28	0.35
Openness	255	−2.56 (1.84)	−2.57 (1.87)	−2.53 (1.77)	0.81	0.81
**Attitude toward psychiatry**						
Mean	254	2.48 (0.76)	2.45 (0.74)	2.53 (0.82)	0.36	0.69
Willingness to complete specialist training in psychiatry	254	1.94 (1.03)	1.90 (0.97)	2.06 (1.17)	0.11	0.45
Level of personal knowledge	256	2.38 (0.78)	2.37 (0.75)	2.40 (0.85)	0.87	0.87
Level of personal interest	256	3.13 (1.07)	3.11 (0.99)	3.18 (1.25)	0.52	0.69
**Stereotype-content model**						
Competence	249	3.68 (0.82)	3.70 (0.81)	3.64 (0.85)	0.93	0.93
Warmth	249	4.11 (0.83)	4.08 (0.80)	4.18 (0.90)	0.24	0.47
**Opening Minds Stigma Scale for Health Care Providers**						
Sum	256	45 (9)	45 (8)	46 (9)	0.62	0.76
Attitudes	256	16.0 (4.2)	15.9 (4.0)	16.2 (4.7)	0.76	0.76
Disclosure	254	14.0 (3.7)	13.9 (3.7)	14.3 (3.7)	0.50	0.76
**Community attitudes to mental illness**						
Authoritarianism	256	2.17 (0.34)	2.17 (0.33)	2.18 (0.39)	0.84	0.98
Benevolence	256	3.92 (0.42)	3.93 (0.40)	3.91 (0.47)	0.98	0.98
Social restrictiveness	256	1.77 (0.39)	1.74 (0.37)	1.82 (0.43)	0.32	0.98
Community mental health ideology	256	3.88 (0.51)	3.89 (0.50)	3.85 (0.54)	0.60	0.98

^a^*n* (%); Mean (SD).^b^Pearson's χ^2^ test; Wilcoxon rank sum test; Welch Two Sample *t*-test.^c^False discovery rate correction for multiple testing.

Our comparisons between the completer (*n* = 183) and non-completer (*n* = 73) groups revealed no significant differences. Interestingly, the expression of the big-five traits of conscientiousness was higher in the completers, but this finding missed the significance level after the FWE correction (*p*_FDR-adjusted_ = 0.20; see Table 2).

### 3.2 Evaluations after finishing the clerkship

As depicted in Table 3 and Supplementary Figure 1A, the evaluation values vary in the upper range, indicating relatively high satisfaction with the psychiatric clerkship. Regarding staff motivation, physicians and psychologists achieved the highest scores for involving medical students in the treatment process (Mean = 2.17 [SD = 1.36]), ahead of occupational therapists, social workers (2.86 [1.72]), and nursing staff (2.92 [1.65]). Among all clerkship evaluation ratings, the items “Conducting face-to-face conversations with patients” ([1.82 1.13]) and “Experience of daily life on the ward…” (2.94 [1.62]) were rated the highest and lowest, respectively. Among the four items that addressed perceived aspects of contact with patients (see Supplementary Figure 1B), “Me and the patient understand each other” (1.56 [0.91]) and “I find the patient difficult to interact with (−1.28 [1.49]) were rated the highest and lowest, respectively.

**Table 3:** Evaluation after the finishing of the clerkship (T2).

**Items**	***N***	***N* = 183^a^**
**Staff motivation**		
mot1: Motivation of doctors and psychologists	180	2.17 (1.36)
mot2: Motivation of nurses	179	2.92 (1.65)
mot3: Motivation of social workers and occupational therapists	153	2.86 (1.72)
**Organization**		
org1: How educational was it to experience daily life on the ward by being always present?	180	2.94 (1.62)
org2: How educational was it to do the patient exams myself?	179	2.22 (1.40)
org3: How educational was it to care for my patients?	176	2.23 (1.41)
org4: How educational was it to follow patients over 2 weeks?	178	3.06 (1.65)
org5: How educational was it to be able to have one-on-one conversations with patients?	180	1.82 (1.13)
**Overall impression**	180	2.74 (1.32)
**Contact with patients**		
con1: The patient took an active part in the discussions with me	181	1.98 (1.12)
con2: I felt comfortable in my interaction with the patient	181	1.78 (1.04)
con3: Me and the patient understand each other	180	1.56 (0.91)
con4: I find the patient difficult to interact with	181	−1.28 (1.49)

^a^Mean (SD).

### 3.3 Effects of the intervention (completer analysis)

**Figure 1:**
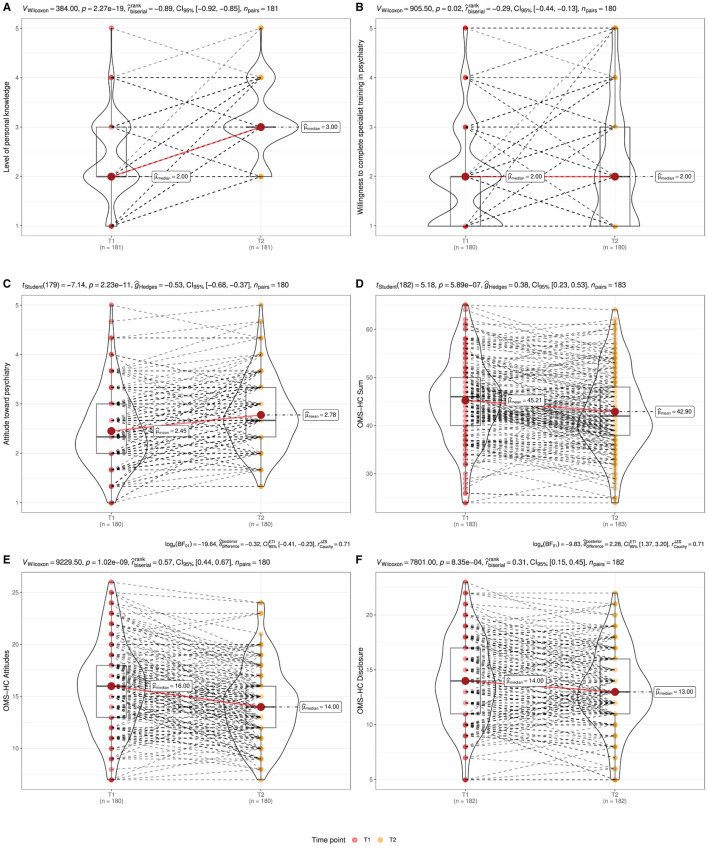
Combined violin-/ boxplots represent stigma reduction after the psychiatry clerkship —as measured using the questionnaire addressing the attitude toward psychiatry (ATP) and the Opening Minds Scale for Healthcare Providers (OMS-HC). Significant higher ratings in ATP **(A)** “personal knowledge”, **(B)** “willingness to complete specialist training in psychiatry”, and **(C)** mean ATP value. Significant lower ratings in OMS-HC **(D)** sum and subscales **(E)** “attitudes” and **(F)** disclosure after the clerkship.

**Figure 2:**
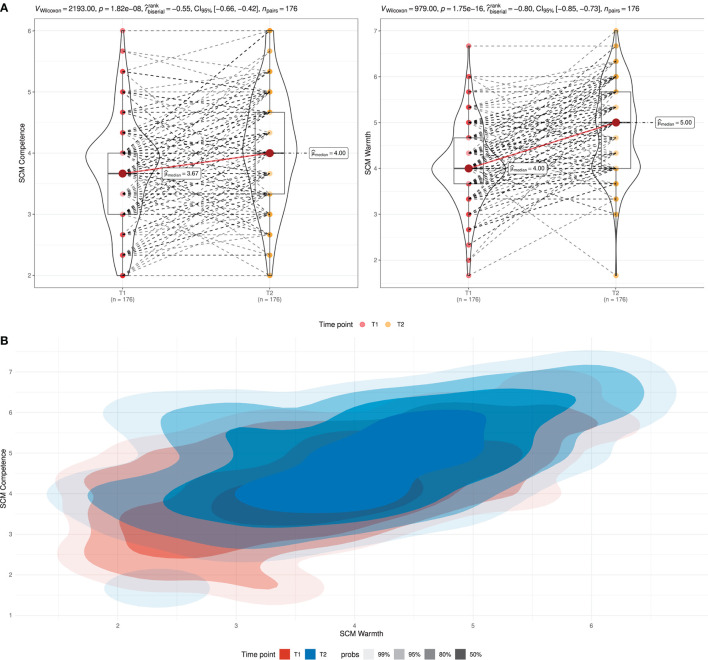
Stigma reduction after the psychiatry clerkship, as measured using the stereotype-content model (SCM). **(A)** Combined violin-/ boxplots represent significant higher ratings in SCM domains “competence” and “warmth” after the clerkship. **(B)** The effect of stigma reduction after the psychiatry clerkship was measured using the stereotype-content model presented in a two-dimensional density plot.

As depicted in Table 4, Figures 1, 2, we measured a significant reduction in stigma after psychiatry clerkship.

**Table 4:** Stigma measurements before (T1) and after (T2) the clerkship: completers only analysis.

**Characteristic**	***N***	**T1, *N* = 183^a^**	**95% CI^b^**	**T2, *N* = 183^a^**	**95% CI^b^**	***p*-value^c^**	***q*-value^d^**
**Attitude toward psychiatry**							
Mean	363	2.45 (0.74)	2.3, 2.6	2.78 (0.69)	2.7, 2.9	< 0.001	< 0.001
Level of personal interest	364	3.11 (0.99)	3.0, 3.3	3.20 (0.92)	3.1, 3.3	0.13	0.19
Level of personal knowledge	364	2.37 (0.75)	2.3, 2.5	3.08 (0.66)	3.0, 3.2	< 0.001	< 0.001
Willingness to complete specialist training in psychiatry	363	1.90 (0.97)	1.8, 2.0	2.06 (1.13)	1.9, 2.2	0.023	0.037
**Stereotype-content model**							
Competence	357	3.70 (0.81)	3.6, 3.8	4.09 (0.86)	4.0, 4.2	< 0.001	< 0.001
Warmth	357	4.08 (0.80)	4.0, 4.2	4.80 (0.91)	4.7, 4.9	< 0.001	< 0.001
**Opening minds stigma scale for healthcare providers**							
Sum	366	45 (8)	44, 46	43 (8)	42, 44	< 0.001	< 0.001
Attitudes	363	15.9 (4.0)	15, 16	14.2 (3.5)	14, 15	< 0.001	< 0.001
Disclosure	365	13.9 (3.7)	13, 14	13.3 (3.5)	13, 14	< 0.001	0.002
**Community attitudes to mental illness**							
Authoritarianism	365	2.17 (0.33)	2.1, 2.2	2.19 (0.35)	2.1, 2.2	0.35	0.38
Benevolence	365	3.93 (0.40)	3.9, 4.0	3.90 (0.44)	3.8, 4.0	0.65	0.65
Social restrictiveness	365	1.74 (0.37)	1.7, 1.8	1.73 (0.46)	1.7, 1.8	0.35	0.38
Community mental health ideology	365	3.89 (0.50)	3.8, 4.0	3.93 (0.52)	3.9, 4.0	0.23	0.29

^a^Mean (SD).^b^CI, confidence interval.^c^Wilcoxon signed rank test with continuity correction; Paired *t*-test.^d^False discovery rate correction for multiple testing.

With respect to the measurement of attitude toward psychiatry, we observed a more positive rating after the clerkship:

- Attitude toward psychiatry (ATP mean: Δ_Mean_ = −0.33; 95% CI [−∞, −0.25], *t*_(179)_ = −7.14, *p* < 0.001; *d*_Cohen_ = −0.53, 95% CI [−∞, −0.40]; see Figure 1C).
- Level of personal knowledge (ATP knowledge: $W=384.00,\;p<0.001;\;{\hat{r}}_{\text{biserial}}^{\text{rank}}=-0.89,\;\text{95\% CI}\;\left[-1.00,-0.86\right]$; see Figure 1A).
- Willingness to complete specialist training in psychiatry (ATP willingness: $W=905.50,\;p=0.037;\;{\hat{r}}_{\text{biserial}}^{\text{rank}}=-0.29,\;\text{95\% CI}\;\left[-1.00,-0.16\right]$; see Figure 1B).

However, we did not measure any significant changes in the level of personal interest in psychiatry (ATP interest), which remained in the neutral range.

Similarly to the measurement of ATP, we observed a significant reduction in the sum and both subscales of the OMS-HC:

- OMS-HC sum (Δ_Mean_ = 2.32, 95% CI [1.58, ∞], *t*_(182)_ = 5.18, *p* < 0.001; *d*_Cohen_ = 0.38, 95% CI [0.26, ∞]; Figure 1D).
- OMS-HC attitudes ($W=9229.50,p<0.001;{\hat{r}}_{\text{biserial}}^{\text{rank}}=0.57,\;\text{95\% CI}\;\left[0.46,1.00\right]$; Figure 1E).
- OMS-HC disclosure ($W=7801.00,p<0.001;{\hat{r}}_{\text{biserial}}^{\text{rank}}=0.31,\;\text{95\% CI}\;\left[0.18,1.00\right]$; Figure 1F).

As depicted in Figure 2, we measured a statistically significant and strong increases in both SCM domains:

- Competence ($W=2193.00,\;p<0.001;{\hat{r}}_{\text{biserial}}^{\text{rank}}=-0.55,\;\text{95\% CI}\;\left[-1.00,-0.44\right]$).
- Warmth ($W=979.00,\;p<0.001;{\hat{r}}_{\text{biserial}}^{\text{rank}}=-0.80,\;\text{95\% CI}\;\left[-1.00,-0.74\right]$).

Visual analysis of the two-dimensional density plot (Figure 2B) demonstrated that there were almost no observations with extreme negative ratings (below −1.5) affecting both SCM domains.

Surprisingly, our pre-vs.-post comparisons in all CAMI subscales did not reveal any significant differences (see Table 4).

### 3.4 Associations between the willingness to complete specialist training in psychiatry and the stigma measurements

#### Before clerkship

**Figure 3:**
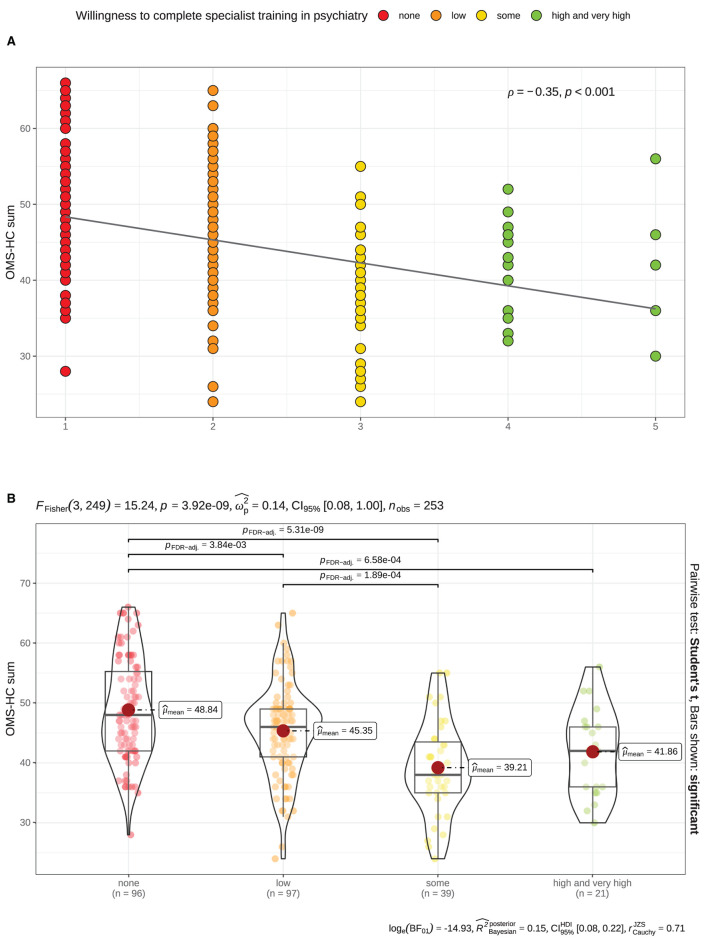
Significant association between stigma expression before the psychiatry clerkship (T1) —as measured using the Opening Minds Scale for Health Care Providers; OMS-HC sum score— and the “willingness to complete specialist training in psychiatry”. **(A)** Scatterplot demonstrates a significant inverse correlation (Spearman). **(B)** Combined violin-/ boxplot demonstrates the differences of the OMS-HC sum scores depending on the level of the “willingness”… before the clerkship. Color coding represents the ratings in “willingness”… at baseline —as measured using a 5-items Likert scale: red Value = 1 [none]; orange Value = 2 [low]; yellow Value = 3 [some]; green Value > 3 [high and very high].

As depicted in the first correlation matrix (Supplementary Figure 3A), all stigma measurements correlated with the willingness to complete psychiatry specialist training after finishing medical school. These correlations demonstrate an association between lower stigma expression and more prominent “willingness…”; the effect size range ranged from low (e.g., OMS-HC disclosure: ρ_Spearman_ = −0.14) to very large (OMS-HC attitudes: ρ_Spearman_ = −0.43). The second most prominent correlation was with the OMS-HC sum, see Figure 3A. Our further analysis used ANOVA to test the differences between subgroups of medical students with willingness “non-present,” “low,” “some,” and “high or very high” showed significant differences between the groups. *Post-hoc*, we observed significant differences between the groups, with the exception of the comparison between students with “some” and “high or very high” rating in the willingness at T1, see Figure 3B.

#### Changes (Δ) after clerkship

**Figure 4:**
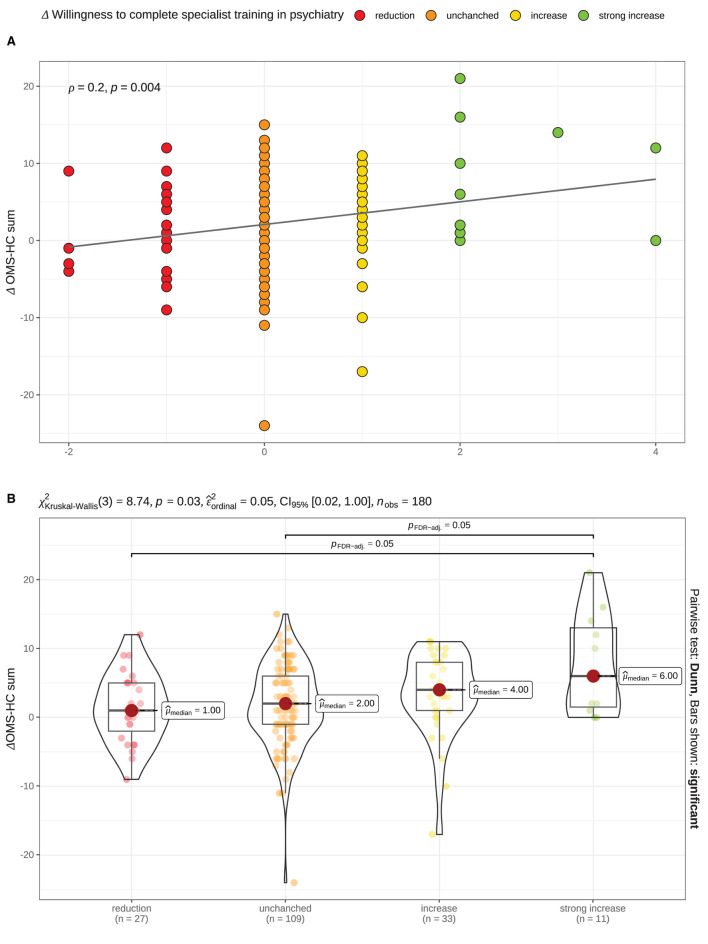
Significant association between degrees of changes in “willingness to complete specialist training in psychiatry” and stigma —as measured using the Opening Minds Scale for Health Care Providers; OMS-HC sum score— after the psychiatry clerkship. **(A)** Scatterplot demonstrates a significant correlation (Spearman). **(B)** Combined violin-/ boxplot demonstrates the differences of the OMS-HC sum scores depending on the degree of the “willingness”… changes after the clerkship. Δ = Value_T2_ − Value_T1_. Color coding represents the degrees of changes in “willingness”… measured using a 5-items Likert scale: red Δ < 0 [reduction]; orange Δ = 0 [unchanged]; yellow Δ = 1 [increase]; green Δ > 1 [strong increase].

As depicted in the second correlation matrix (Supplementary Figure 3B), the increments of only two stigma measurements (Δ OMS-HC sum and the subscale attitudes) correlated with the increment willingness to complete specialist training in psychiatry. These direct correlations demonstrate an association between a more prominent stigma reduction (measured using OMS-HC and attitudes) and a more prominent increase in “willingness…”; the effect sizes were low (both ρ_Spearman_ = 0.2). The correlation with the OMS-HC sum is visualized in Figure 4A.

Our further analysis used ANOVA to test the differences between subgroups of medical students whose rating of the “willingness…” decreased, stayed unchanged, increased “some” and “high or very high” willingness showed significant differences between groups. *Post-hoc*, we observed that the reduction in the OMS-HC sum after the clerkship was significantly higher in students who reported a strong increase (Likert ≥2) compared to those whose “willingness…” remained unchanged or decreased after the clerkship (see Figure 3B).

## 4 Discussion

In our cohort, we observed that completion of the training was associated with a reduction in stigmatizing attitudes held by medical students toward mental disorders and psychiatry in general. Furthermore, their average willingness to become a psychiatrist in the future was reinforced in association after the clerkship in psychiatry, with a positive correlation with the degree of reduction in stigma.

We measured stigma using the stereotype-content model (36), in which both domains “warmth” and “competence” were estimated using a Likert scale, we observed that the ratings in the domain “warmth” were marginally positive and for the “competence” lightly negative at the baseline. Although this pattern might initially appear to be an expression of the healthcare professionals' social awareness, prior publications indicate its negative connotation (45, 46). This SCM pattern describes a condescending relationship with a group of benign subordinates, eliciting pity and sympathy. This attitude implies an inequality, suggesting it as a form of stigmatization (46).

After the clerkship, we observed highly significant changes in absolute Likert scale scores for both domains, as consistent with our *a priori* hypothesis. However, the increase in “warmth” was more prominent than in “competence”. Thus, the baseline pattern “warm but incompetent” remained. A possible explanation might be that the clerkship gave students the opportunity to learn that psychiatric patients are less dangerous than they expected and may thus contribute—by reducing anxiety—to higher “warmth” ratings, as previously supposed (45, 47). Simultaneously, impaired cognition and social functioning deficits accompanying severe mental illnesses can in principle hinder higher ratings in the SCM domain “competence”, and thus lead to the persistence of the baseline pattern after the clerkship. However, we interpret these results as a correlate of stigma reduction, one reason being the prominent reduction in the high levels of expressed stigmatization—and corresponding to the lowest ratings in both SCM domains simultaneously—, as depicted using the two-dimensional density plot in Figure 2.

For a proper interpretation of our results, it is relevant to mention that baseline ratings in both SCM domains were relatively high compared to previously published data (48), which argues for a rather low initial level of stigmatization in our cohort. Therefore, a possible floor effect that decreases the strength of stigma reduction should be considered. Similarly to baseline ratings in the SCM domains, we observed a comparatively low baseline stigma expression measured with the OMS-HC, a tool to test stigmatization among medical providers (41). However, we still observed a significant reduction in the mean values of the OMS-HC sum and its subscale “attitudes” scores after the clerkship, in support of our *a priori* hypothesis. These findings are in line with the recent observation in a cohort of Canadian medical students (49).

Furthermore, we observed a reduction in the OMS-HC subscale “disclosure”, which explicitly addresses the attitude toward personal mental health of participants, and which represents “self-stigma” tendencies among healthcare professionals (50). Healthcare providers may associate their professional image with alleged “invulnerability” and “omnipotence”, and may thus lead to self-stigmatization due to a mismatch with an irrationally high benchmark reference; they might then avoid help seeking and delay adequate support (51). This may be a reason for the increase in burnout rates among medical students (52, 53). Our findings indicate that a psychiatry clerkship may reduce “self-stigma” and, therefore, can contribute to better mental health among medical students and healthcare providers.

The other stigma measurement tool we used was the Community Attitudes to Mental Illness (CAMI) scale, that measures social stigma (42, 54). Although we observed several significant correlations between baseline values or increments in CAMI subscales and other stigma measurements (see Supplementary Figure 3), no significant effects of clerkship could be found in our cohort. Since the mean values of the CAMI subscales “authoritarianism” and “social restriction” were lower in our cohort than in previously published results (55, 56), we speculate that the lack of clerkship effect could also be due to a “floor/ceiling effect” associated with a comparatively low baseline level of stigma in the current cohort, as recently assumed (27). Furthermore, the lack of significant intervention effects may also be associated with limitations of the CAMI questionnaire, which was originally designed to be applied in the general population, but which is not specific to healthcare professionals. Moreover, a recent systemic review has revealed that, since the first introduction of CAMI in 1981, only few longitudinal studies with CAMI have been published, and these possibly indicate that this measure exhibits limited temporal stability (54).

In further analysis, we compared self-assessed levels of personal knowledge, interest, and willingness to work as a psychiatrist in the future before and after the clerkship. We hypothesized that these items would increase significantly after the clerkship. At baseline, the mean values of the Likert scale for “interest” were rated neutral, “knowledge” neutral to negative and “willingness” negative. After the clerkship, the levels of “knowledge” and “willingness” increased significantly. However, the mean values for “willingness” remained in the negative range of the Likert scale. Furthermore, there were no significant changes in personal “interest” after the clerkship. Since our cohort's baseline level of “interest” was relatively high, this could explain the lack of a significant increase, and corresponds to previous findings (17, 57). The most prominent growth was measured in the subjective evaluation of “knowledge”, which is not surprising, as this is the primary objective of the intervention.

In addition, we found an association between the willingness to complete psychiatric specialist training and the level of stigmatization measured using the OMS-HC scale, which demonstrated lower stigma in students who rated their “willingness” as above average; see Figure 3. Furthermore, we observed a similar trend in changes in the rating after the clerkship. As seen in Figure 4, growth in “willingness” was associated with a more prominent reduction in stigma. These findings align with our *a priori* hypothesis and the previously published results (17).

### 4.1 Limitations

In general, it is necessary to take into account that the fundamental methodical challenges of questionnaires—e.g., biasing due to “social desirability” and “tendency to the center”—limit the interpretation of current findings. In addition, Likert scales are inherently limited by the specification of response options and the restriction of multiple responses. Specifically for the current cohort, the relatively low expression of stigma before training must be regarded as a limitation. As mentioned above, the possible floor and ceiling effect may potentially lead to underestimation of the intervention effects and therefore need to be taken into account during the interpretation of the results. The direct interaction between medical students and patients is an essential component of our training. However, no data on the diagnoses and severity of mental conditions by patients were evaluated, although these may have an impact on stigma, and may enhance stigma after interactions with very severely ill patients [e.g., (45)]. Similarly, only limited differentiation was possible of the influencing characteristics of the staff or the previously completed clerkship. Finally, the lack of a control group should be considered as another limitation.

Future research should be aware of the limitations mentioned here and continue to address the role of direct student-patient interaction in stigma reduction, compare clerkship programs across various medical schools and countries, and consider the effects of patients' characteristics (e.g., diagnoses, severity of symptoms, social functioning level) and the perceived teaching skills, motivation, and personality profiles of academic mentors. Additionally, future studies should address the development and implementation of novel interventions that help reduce stigma (58, 59).

## Conclusion

Prior research underscores the widespread nature of stigma associated with severe mental illnesses and its detrimental impact on access to adequate healthcare. Stigma can lead to suboptimal treatment, shortened life expectancy, and indirect discrimination against patients. Our current findings provide additional evidence that direct student-patient interaction is highly effective in reducing stigma, as shown by the significant reduction in prejudice observed among medical students after a psychiatry training program. Furthermore, we identified a correlation between reduced stigma and increased willingness to pursue a psychiatric residency after medical school. These results align with previous studies, and emphasize the importance of addressing stigma in healthcare-related educational programs. Therefore, we advocate the incorporation of components of direct interaction in medical education, in order to combat stigma and improve patient outcomes.

## Data availability statement

The raw data supporting the conclusions of this article will be made available by the authors, without undue reservation.

## Ethics statement

The current study was approved by Marburg University Ethics Committee and conducted in accordance with the local legislation and institutional requirements. The participants provided their written informed consent to participate in this study.

## Author contributions

MZ: Conceptualization, Data curation, Formal analysis, Methodology, Visualization, Writing—original draft. SK: Conceptualization, Investigation, Methodology, Project administration, Writing—review & editing. YY: Data curation, Formal analysis, Methodology, Writing—review & editing. WL: Conceptualization, Data curation, Formal analysis, Methodology, Validation, Writing—review & editing. UW: Conceptualization, Supervision, Writing—review & editing. TK: Conceptualization, Project administration, Resources, Supervision, Writing—review & editing.
