# Supplementary material for: The effects of a psychiatric clerkship on stigmatizing attitudes toward mental disorders as held by German medical students
Source: Front Psychiatry. 2023 Dec 8;14:1306403. doi: 10.3389/fpsyt.2023.1306403 (PMC10748402; doi:10.3389/fpsyt.2023.1306403)
Supplement: Supplementary file 1 [file Data_Sheet_1.pdf]

Supplementary Materials

1 AD METHODS

1.1 Measuring tools

1.1.1 Attitude towards Psychiatry

At T1 and T2, we use a 5-point Likert scale to assess personal levels of interest, knowledge, and willingness to complete specialist training in psychiatry after finishing medical school. The Likert scale value of 1 represents the lowest, 5 the highest, and 3 the average levels, respectively. Previous research shows that these items appear suitable for measuring students' attitudes toward psychiatry (ATP) and people with mental illness (Cutler et al., 2009; Takeuchi and Sakagami, 2018). Additionally, we calculated the means of the three items, in order to build a putative score describing a general attitude toward psychiatry.

1.1.2 Stereotype-Content Model

The stereotype-content model (SCM) assumes two basic domains of social perception: **warmth** and **competence** (Fiske et al., 2002). These two attributions are basic survival-critical evolutionary assessments in social interaction because they distinguish between a "friend" or "enemy / competitor" , who were potentially "dangerous" or "harmless". They evoke uniformly negative or positive emotions in the observer (Fiske et al., 2002, 2007). Furthermore, this model may explain the ambivalent stigmatizing attitudes towards people with mental illness, for example, "highly competent but very cold" (Sadler et al., 2012). This model has recently been used to identify stereotypes about immigrant populations in Germany (Froehlich and Schulte, 2019). In the current work, we asked participants to rate how warm, sympathetic and good-natured they were and how competent, competitive and independent they perceived patients with mental illness. The answers were given according to a 7-point Likert scale ranging from 1 ("not at all") to 7 ("completely"). Four is a neutral evaluation, and values higher or lower than four represent positive or negative evaluations, respectively (Jenaro Río et al., 2018). The means of the two three-item clusters formed putative scores describing individual ratings of perceived warmth and competence in patients with mental illness.

1.1.3 Opening Minds Scale for Health Care Providers

The Opening Minds scale for Health Care Providers (OMS-HC) was developed in 2011 to measure stigma among healthcare providers towards people with mental illness (Kassam et al., 2012). The questionnaire contains 20 items, each with a 5-point Likert scale. Seven items (3, 8, 9, 10, 11, 15, 19) are in inverse alignment. The **total sum score** can vary from 20 to 100. A higher score indicates a higher expression of stigma. Furthermore, the authors of the OMS-HC provide two subscales for further differentiation: **Attitudes** of medical providers toward people with mental illness (subscale attitudes; sum of seven items) and **Disclosure** of potential personal mental health problems (subscale disclosure; the sum of five items; Kassam et al., 2012).

1.1.4 Community Attitudes Toward the Mentally Ill

The Community Attitudes Toward the Mentally Ill (CAMI) is a further stigma measuring tool, with four highly reliable subscales (Taylor and Dear, 1981; Lien et al., 2019; O' Connor et al., 2013). The items use a 5-point Likert scale, ranging from 1 ("strongly agree") to 3 ("neutral") and 5 ("strongly disagree"). Four subscales reflect different aspects of public attitude:

- The **authoritarianism** subscale uses questions such as: "Do mentally ill people, like children, need external control?" or "People should be hospitalized at the slightest sign of psychiatric symptoms".
- The **benevolence** subscale refers to society having a responsibility towards people with mental illness and, for example, "more tax

money should be allocated to care or that society should be more tolerant of the mentally ill" and "more should be done to provide psychiatric care".

- The subscale **social restrictiveness** refers to the attitude toward people with mental illness, for example, that they are dangerous, should be avoided, and should be isolated from the rest of society.
- The subscale **community mental health ideology** asks, for example, whether psychiatric hospitals should be in the neighborhood or whether participation in public life is the best therapy for people with mental illness.

Each subscale can achieve a maximum score of 50. A total score is not helpful as interpretation of the individual subscales may conflict. Originally, the CAMI scale contained 40 items. However, we decided to remove seven items from the mental health community ideology subscale to avoid redundancies with other measuring tools and to make it easier for the participants to complete the survey.

1.1.5 Clerkship Evaluation Questionnaire

On the last day of the clerkship (follow-up or T2), we evaluated the individual perception of distant aspects of the training using a 7-point Likert scale ranging from -3 ("very poor") to 0 ("neutral") and 3 ("very good"). In addition to an overall impression (item: "overall"), we divided the questionnaire into three subscales:

- The subscale **staff motivation** contains questions that evaluate the perceived participation of physicians and psychologists (item: mot1), nurses (item: mot2), as well as occupational therapists and social workers (item: mot3), in terms of the supervision of the teaching of medical students.
- The subscale **structural quality of training** contains five items that address the perceived importance of different aspects of the clerkship for the personal learning outcome (items: org1-5).
- The subscale **contact with patients** included the four items that measure students' perception of the interaction and conversation during their contact with patients they met during their clerkship (items: con1-4).

For an exact phrasing of the items, see Table 3 in the main manuscript.

1.1.6 Big Five Personality Traits

The "Big Five" personality traits are openness to experience, conscientiousness, extraversion, agreeableness, and neuroticism. Each trait represents a continuum, and individuals can fall anywhere on the continuum for each trait. Evidence suggests that personality is highly stable during adulthood, although minor changes may be possible (Goldberg, 1990; Yuan et al., 2018). In our questionnaire, the personality poles were selected from the Ten Items Personality Inventory (TIPI; Nunes et al., 2018).

1.1.7 Level-of-Contact Report (LOCR)

The questionnaire for measuring various contact experiences with people with mental illness initially contains 12 questions that can be answered with "yes" or "no". The highest score (12) indicates that the respondent has a mental illness. The lowest score (1) indicates that the respondent had never observed a person who might have had a mental illness (Holmes et al., 1999). In the present work, we used a ten items scale, whereas the items "I have never observed a person who had a serious mental illness" and "My job involves providing services to people with mental illness" were omitted. Thus, this survey yields a maximum score of 70.

1.1.8 Other Surveyed Information

In addition, we recorded age, gender, semester assignment, number of days of clerkship, and whether participants completed the clerkship in child psychiatry before attending our training.

2 SUPPLEMENTARY FIGURES

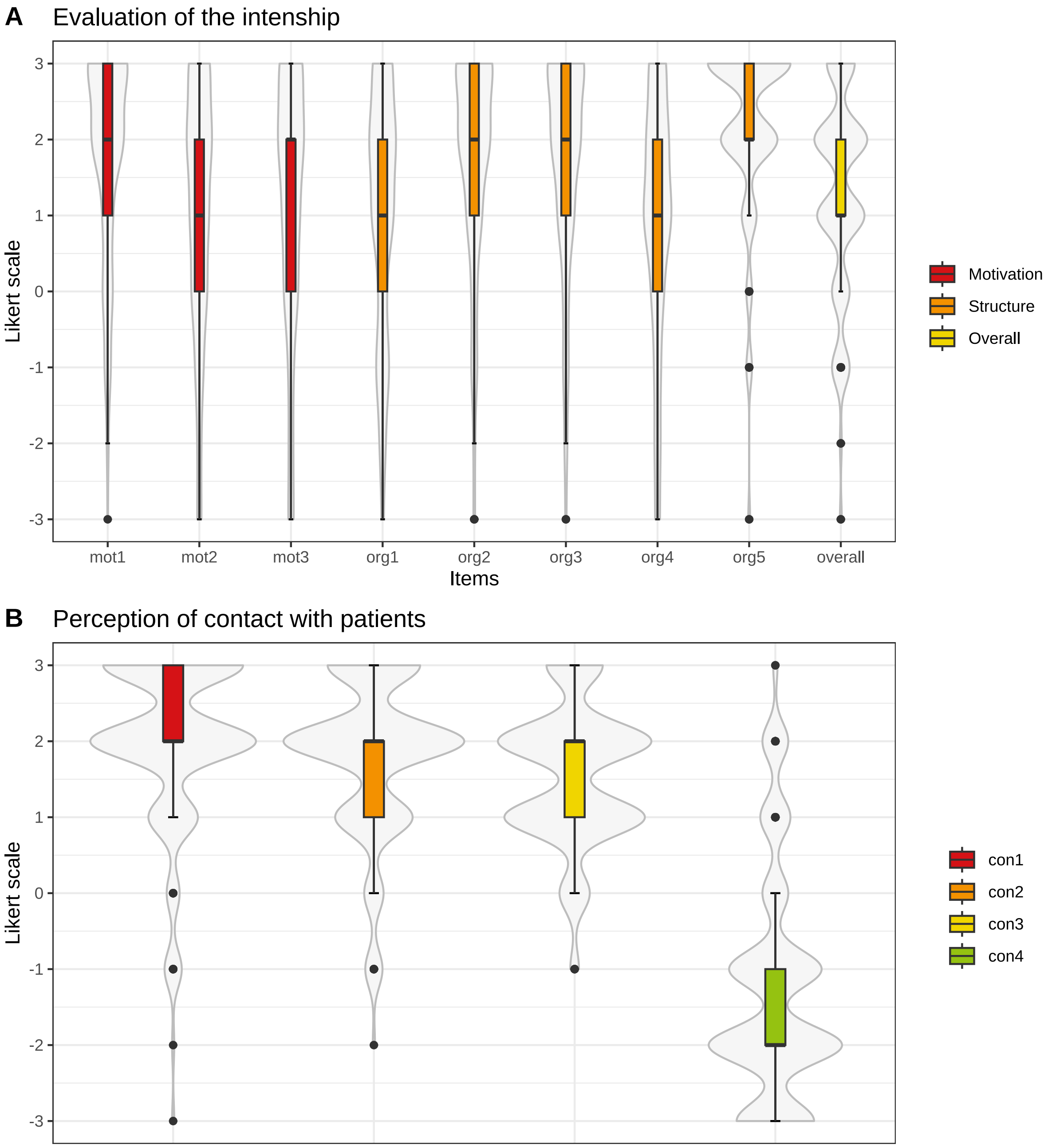

Figure S1. Evaluation after the clerkship (T2): **(A)** The combined violin-/ boxplot demonstrates nine items measuring the perception of staff motivation (mot1-3), structural organization (org1-5), and the overall impression (overall) of the clerkship. **(B)** The combined violin-/ boxplot demonstrates four items measuring the perception of patient contact during the clerkship (con1-4). The variable con4 is in a negative alignment. In the main manuscript, Table 3 provides the exact wording of the items mot1-3, org1-5, and con1-4.

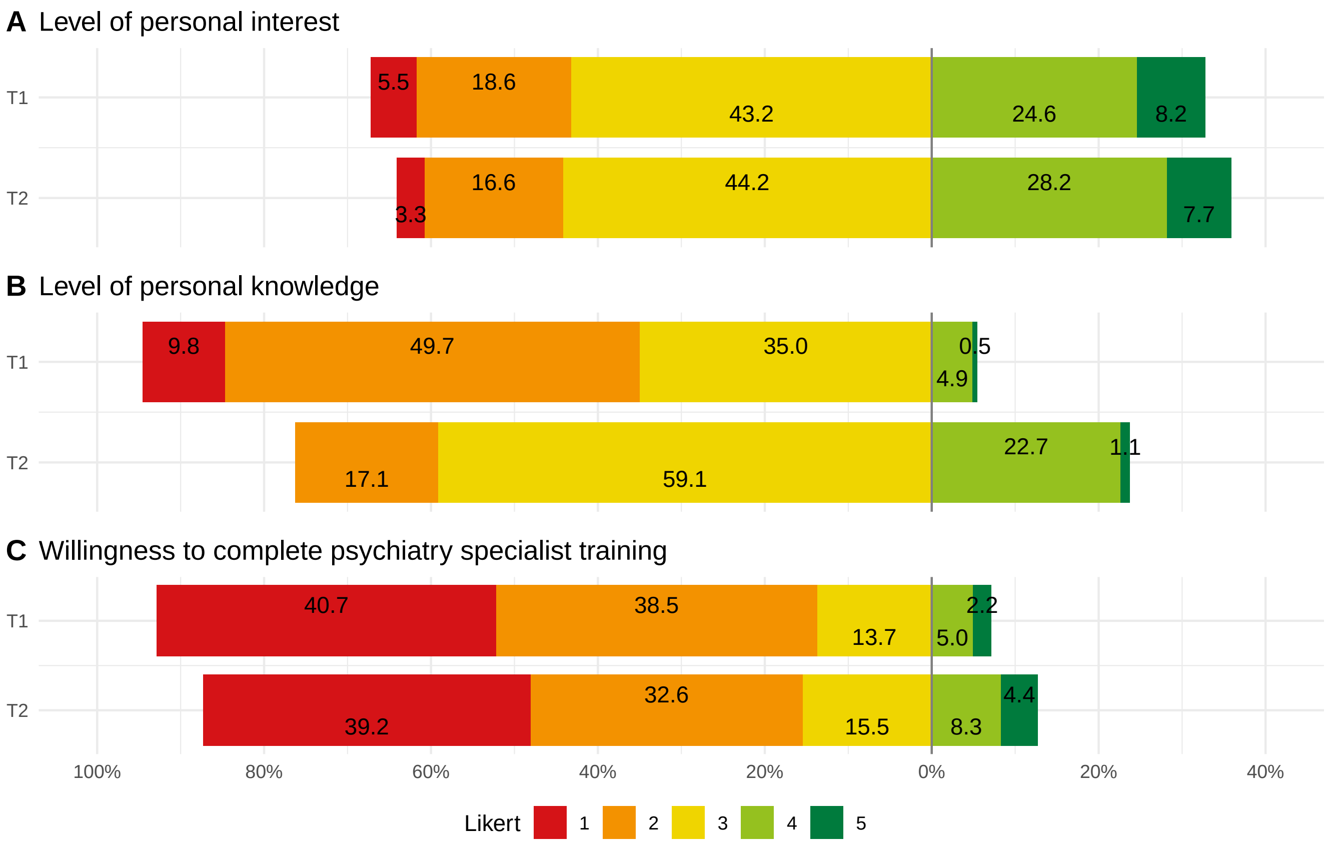

Figure S2. Measurements of the attitude towards psychiatry using a 5-item Likert scale and including the questions about perceived levels of personal **(A)** "interest", **(B)** "knowledge", and **(C)** "willingness to complete psychiatry specialist training" before (T1) and after our internship (T2). 5-item Likert scale: "1" represents the lowest ranking and "5" the highest ranking. Numbers inside the color bars represent the percentage of participants.

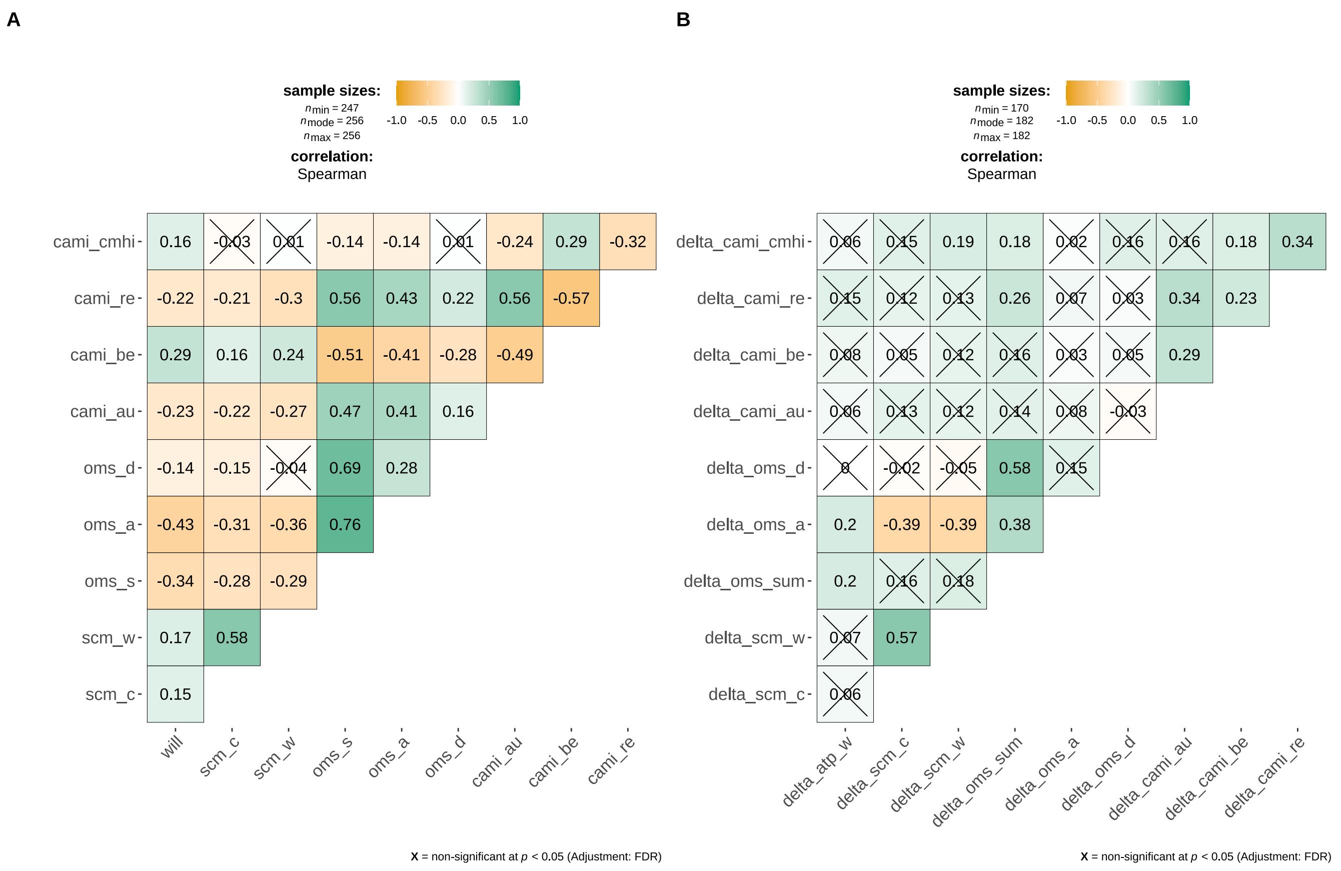

Figure S3. Correlation matrixes describing the associations (**A**) between the "willingness to complete specialist training in psychiatry" and stigma measuring tools before and (**B**) their changes after the clerkship in psychiatry.

REFERENCES

Cutler JL, Harding KJ, Mozian SA, Wright LL, Pica AG, Masters SR, et al. Discrediting the notion “working with ‘crazies’ will make you ‘crazy”’: addressing stigma and enhancing empathy in medical student education. *Advances in Health Sciences Education* **14** (2009) 487–502. doi:10.1007/s10459-008-9132-4.

Takeuchi J, Sakagami Y. Stigma among international students is associated with knowledge of mental illness. *Nagoya Journal of Medical Science* **80** (2018) 367–378. doi:10.18999/nagjms.80.3.367.

Fiske ST, Cuddy AJC, Glick P, Xu J. A model of (often mixed) stereotype content: Competence and warmth respectively follow from perceived status and competition. *Journal of Personality and Social Psychology* **82** (2002) 878–902. doi: 10.1037/0022-3514.82.6.878.

Fiske ST, Cuddy AJ, Glick P. Universal dimensions of social cognition: warmth and competence. *Trends in Cognitive Sciences* **11** (2007) 77–83. doi:10.1016/j.tics.2006.11.005.

Sadler MS, Meagor EL, Kaye KE. Stereotypes of mental disorders differ in competence and warmth. *Social Science & Medicine* **74** (2012) 915–922. doi:10.1016/j.socscimed.2011.12.019. Part Special Issue: Migration, ’illegality’, and health: Mapping embodied vulnerability and debating health-related deservingness.

Froehlich L, Schulte I. Warmth and competence stereotypes about immigrant groups in germany. *PLOS ONE* **14** (2019) 1–17. doi:10.1371/journal.pone.0223103.

Jenaro Río C, Flores Robaina N, López Lucas J. Utilización de la técnica del diferencial semántico para evaluar estereotipos hacia personas con discapacidades: La relevancia de la calidez y competencia. *Universitas Psychologica* **17** (2018) 1–12. doi:10.11144/Javeriana.upsy17-4.usdt.

Kassam A, Papish A, Modgill G, Patten S. The development and psychometric properties of a new scale to measure mental illness related stigma by health care providers: The opening minds scale for health care providers (oms-hc). *BMC Psychiatry* **12** (2012) 62. doi:10.1186/1471-244X-12-62.

Taylor SM, Dear MJ. Scaling Community Attitudes Toward the Mentally Ill. *Schizophrenia Bulletin* **7** (1981) 225–240. doi:10.1093/schbul/7.2.225.

Lien YY, Lin HS, Tsai CH, Lien YJ, Wu TT. Changes in attitudes toward mental illness in healthcare professionals and students. *International Journal of Environmental Research and Public Health* **16** (2019). doi:10.3390/ijerph16234655.

O’ Connor K, Brennan D, O’ Loughlin K, Wilson L, Pillay D, Clarke M, et al. Attitudes towards patients with mental illness in irish medical students. *Irish Journal of Medical Science* **182** (2013) 679–685. doi:10.1007/s11845-013-0955-5.

Goldberg LR. An alternative ”description of personality”: The big-five factor structure. *Journal of Personality and Social Psychology* **59** (1990) 1216–1229. doi:10.1037/0022-3514.59.6.1216.

Yuan Q, Seow E, Abdin E, Chua BY, Ong HL, Samari E, et al. Direct and moderating effects of personality on stigma towards mental illness. *BMC Psychiatry* **18** (2018) 358. doi:10.1186/s12888-018-1932-3.

Nunes A, Limpo T, Lima CF, Castro SL. Short scales for the assessment of personality traits: Development and validation of the portuguese ten-item personality inventory (tipi). *Frontiers in Psychology* **9** (2018). doi:10.3389/fpsyg.2018.00461.

Holmes EP, Corrigan PW, Williams P, Canar J, Kubiak MA. Changing Attitudes About Schizophrenia. *Schizophrenia Bulletin* **25** (1999) 447–456. doi:10.1093/oxfordjournals.schbul.a033392.
